# The Shock of the New: Progress in Schizophrenia Genomics

**DOI:** 10.2174/138920211797904089

**Published:** 2011-11

**Authors:** Susan Moore, Eric Kelleher, Aiden Corvin

**Affiliations:** 1 Neuropsychiatric Genetics Research Group, Department of Psychiatry and Institute of Molecular Medicine, Trinity College, Dublin, Ireland

**Keywords:** Copy Number Variation, DNA variants, neurodevelopmental disorders, psychosis, schizophrenia.

## Abstract

A growing list of common and rare genetic risk variants are being implicated in schizophrenia susceptibility. As with other complex genetic disorders most of the variance in genetic risk is still to be attributed. What can be learned from progress to date? The available data challenges how we conceptualize schizophrenia and suggests strong aetiological links with other psychiatric and developmental disorders. With the identification of rare copy number risk variants implicating specific genes (e.g. VIPR2 and NRXN1) it is increasingly possible to investigate molecular aetiology in patient subgroups to establish whether schizophrenia represents one or many different disease processes. This review summarizes recent research progress and suggests how the tools of modern genomics and neuroscience can be applied to best understand this devastating disorder.

## INTRODUCTION

The ‘shock of the new’ referred to a reflexive, premature and generally negative response provoked by the emergence of modern art [1]. Inevitably, with time, distance and perspective a more balanced consensus evolved as it does for other ‘new’ things. The early genomic data for schizophrenia provide a fascinating parallel where many responses have been prematurely negative [2] or focussed on what genome-wide association studies (GWAS) *didn’t* find (the ‘missing heritability debate’) [3] rather than on the many novel, interesting findings that are emerging. 

We believe that genomic and other research tools are available to help us come to grips with these findings. Translating them into pathophysiological insights will take time, but is important for several reasons. The onset of schizophrenia is typically in early adulthood, but the evolution and severity of symptoms, and course of illness are variable despite modern treatments. Schizophrenia is a public health problem as approximately 1% of the adult population are affected and life expectancy is reduced by an average of 20-25 years [4]. Because schizophrenia is substantially heritable, the search for risk genes is not new, but has faced significant obstacles. 

**Fig. (1):**
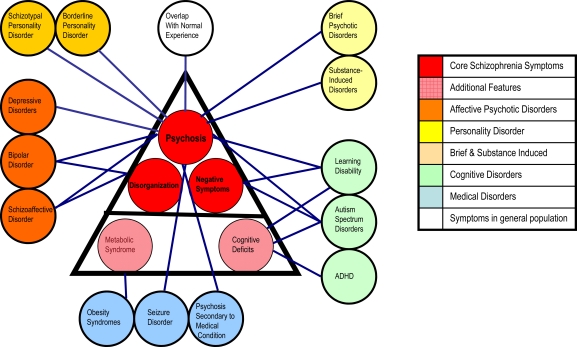
Within the smaller triangle are the core symptom domains of schizophrenia. The larger triangle includes key comorbidities. Each of the external circles represents other psychiatric disorders, medical disorders and the lines indicate how they overlap with specific features of schizophrenia.

Schizophrenia is diagnosed clinically based on a triad of symptom domains (DSM-IV) [5]. One of these symptom groups, psychosis (delusions and hallucinations), is almost ubiquitous during illness episodes. The second, involving disorganization of thinking and behavior, is also strongly associated with psychotic episodes. The final group of ‘negative’ symptoms represents a loss of social function and volition and is more insidious and less amenable to current therapeutics. Many patients are only ever affected in one or two of these domains and within domains, symptoms are also heterogeneous. Although clinically useful, it is uncertain how this diagnosis maps to underlying biology. The extent to which these symptom groups overlap with other psychiatric disorders, medical disorders [6], and even normal human experience [7] is underappreciated but striking (Fig. **1**).

One implication is that psychosis may be an endpoint for many different pathological processes and even a variant of normal human experience in states of stress or restricted consciousness. Social, behavioural and cognitive abnormalities, which also feature in schizophrenia, overlap prominently with other disorders including learning disability and autistic spectrum disorders [8]. This symptom overlap causes ongoing controversy about how the clinical boundaries of the disorder should be drawn [9]. At the same time, a new and more radical view of schizophrenia, based on modern genomics is emerging. Many of the early findings detailed below have been surprising, even shocking, but (with the benefit of hindsight) should not have been entirely unexpected. 

## 1 SCHIZOPHRENIA THE PHENOTYPE

Given the rich genetic epidemiological literature, genetics has long offered the promise of insight into the molecular mechanisms involved in schizophrenia risk. Estimates based on twin-data suggest substantial heritability (h2~0.80-0.85) [10] although many patients have no first-degree relatives with the disorder. Model fitting of twin data indicates that schizophrenia overlaps with another major psychotic condition, bipolar disorder [11]. In fact, data from high-risk studies of the offspring of mothers with schizophrenia suggest that this liability extends to include other psychotic disorders and related personality disorders, termed the ‘schizophrenia spectrum disorders’ [12]. This overlap is not reflected in the early molecular genetics literature for several reasons. Schizophrenia is a clinical diagnosis and in some cases it can be difficult to reach a clear diagnosis, for example in cases with mixed psychotic and affective symptoms [13]. Because of concern about misclassification errors, these types of cases were excluded from genetics analysis. Researchers were also concerned that extending studies beyond the established heritable core diagnosis would reduce study power by including cases with less clear genetic aetiology. 

A remarkable exception to this orthodoxy involved the mapping of the gene Disrupted-in-Schizophrenia-1 (DISC1), which was identified in a large Scottish kindred. A balanced translocation between chromosome 1 and 11 strongly co-segregates with mental disorder in this family. The index case had a diagnosis of conduct disorder and within the family, 18 of 29 (70%) translocation carriers had a major mental disorder (including schizophrenia, bipolar disorder or major depressive disorder), whereas none of the non translocation carriers had such a diagnosis [14]. More than three decades of follow-up in the family indicates that this mutation has a large effect on liability to both schizophrenia and mood disorder in carriers. Recent large-scale epidemiological studies across disorders, demonstrate conclusively that this is a more general phenomenon. In a study of more than 9 million Swedish individuals, Lichtenstein and colleagues [15] confirmed that first degree relatives of probands with either schizophrenia or bipolar disorder are at increased risk of both disorders. The extent of shared liability extends to increased risk of autism [16] and a broad range of mental disorders in relatives of schizophrenia patients [17]. Rates of schizophrenia are also known to be three times higher in people with intellectual disability. Collectively, this data suggests that genetic liability represented by schizophrenia substantially overlaps with other psychiatric and neurodevelopmental disorders. 

## 2 WHAT IS THE GENETIC MODEL?

Based on genetic epidemiological data of risk in different classes of relatives it was suggested that several (or more) risk genes interacted with each other and environmental risk to cause schizophrenia [18]. This model, framed the common disease common variant (CDCV) hypothesis but was challenged by an opposing view that susceptibility involved the influence of rare genetic variants, the common disease rare variant (CDRV) hypothesis. An added dimension to the model is whether schizophrenia represents a single disease entity or many different disease processes or molecular mechanisms; a rare disease rare variant (RDRV) model. Only in the last few years have the molecular methods required to begin testing these models become available; what is emerging is a genetic architecture involving both common and rare risk variation. 

**Fig. (2):**
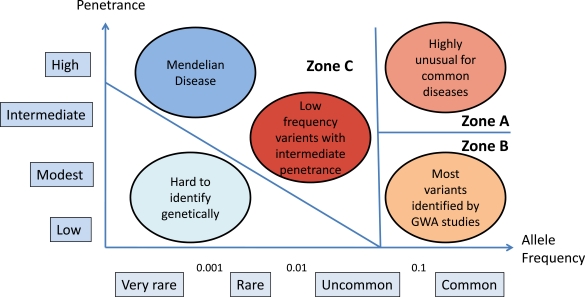
Genetic models for disease. Zone A showing common variants of high penetrance. Genome wide association studies have identified common, low penetrance, risk variants as demonstrated in Zone B. Zone C shows rare risk variants which will be more readily detected by whole genome sequencing. (Adapted from McCarthy *et al.*, 2008 [19]).

Early molecular studies, using linkage and candidate gene association studies (Zone A in Fig. **2**), excluded large, common single gene effects. The recent genome-wide association studies (GWAS) discussed in the next section have started to confirm some common risk variants of modest effect (Zone B in Fig. **2**). From studies of copy number variation we know that examples like the DISC1 translocation, or 22q11.2 deletion syndrome (discussed below) are not unique oddities within ‘mainstream’ schizophrenia. A growing number of rare variants are being discovered and large-scale genome sequencing will allow more comprehensive investigation for rare risk variants (Zone C in Fig. **2**). It remains an open question as to whether the risk variants identified contribute to one or many different molecular mechanisms and represent one or many diseases. 

### 2.1 Common Variant Common Disorder

In the pre-genome era, linkage and candidate gene studies provided a relatively meager return of potential susceptibility loci for schizophrenia and putative candidate genes. This list of candidate genes (including Dysbindin and Neuregulin-1) has received some degree of statistical support. A more definitive list may be found on the SZGene database [20]. As these variants are in some cases poorly assayed by the platforms used for the larger genome-wide association studies (GWAS) detailed below, their status remains equivocal [21]. 

A series of (GWAS) [22-25] and a large meta-analysis of GWAS data, conducted through the Psychiatric GWAS Consortium (PGC), have provided genome-wide significant evidence for at least nine susceptibility loci as demonstrated in Table **1**. 

As has been the consistent theme across common disorders, initial schizophrenia GWAS findings explain only a modest proportion of the variance in susceptibility. A significant proportion of the remainder may involve a polygenic component including hundreds, if not thousands, of common alleles of small effect. Using a polygene score method, the International Schizophrenia Consortium [23] identified substantial overlap in common putative risk alleles of small effect across both schizophrenia and bipolar samples and estimated that these explained at least one-third of total variation in liability. From the emerging GWAS data, many of the associated loci appear to confer liability to both schizophrenia and bipolar disorder [27].

### 2.2. Rare Variants and Schizophrenia

The 22q11.2 deletion syndrome (22q11.2DS; also known as velo-cardio-facial syndrome (VCFS)) is caused by the most common large microdeletion in the human genome and has an incidence of 1 in ~4000 live births. The phenotype is highly variable and can affect multiple organs and tissues, but carriers have a 30-fold increased risk of schizophrenia [28]. An increased, if less substantial risk of schizophrenia is reported in Marfan syndrome [29] and conversely a reduced risk is reported in Down syndrome [8]. Until relatively recently these findings have been seen as novel curiosities that make a limited contribution to what is a relatively common disorder. 

As we became able to detect submicroscopic deletions and duplications in the human genome [30-31] and discovered these to be more common than expected, this has changed. A seminal paper by Walsh and colleagues [32] identified an increased rate of novel deletions and duplications of genes in schizophrenia cases, particularly young onset cases. These mutations were reported to disproportionately disrupt signaling networks controlling neurodevelopment. Two large consortia studies identified association with copy number change at chromosome 1q21.1 and deletions of chromosome15q13.3 [33-34]. Subsequent studies have reported evidence for association with more CNVs implicating 2p16.3, 3q29 and 15q11.2 deletions and duplications of 7q36.3 and 16p11.2. Relative to the common SNP variants these mutations are rare and cumulatively they involve 2-3% of cases, so far [35]. Many of these span many genes, but the 2p16.3 and 7q36 loci implicate specific genes, *NRXN1* and *VIPR2* respectively, which bring them sharply into view for further investigation. 

Unexpectedly, all of these CNVs confer susceptibility to a range of other developmental phenotypes including mental retardation, autism, ADHD, seizure disorders and obesity [36-39]. As an example, the 15q13.3 increases risk for a wide range of clinical features including schizophrenia, autism, seizure disorder, learning disability and cardiac malformations, but a subset of carriers have no discernable clinical findings [40]. By contrast, CNVs appear to be less common in bipolar disorder than in control populations [41]. This is not simply related to the size of the genomic region; at the VIPR2 locus, implicated in schizophenia but also autism cases, the overlapping copy variable region was localized to exons 3 and 4 of the gene [42]. For each of these loci further studies are required to identify the range of phenotypic expression that exists and the disease penetrances. The form of phenotype expression may reflect the influence of other genetic (e.g. common or rare variants) or environmental factors, or other stochastic effects during neurodevelopment. 

## 3 DISCUSSION

Genomic data is re-shaping our understanding of schizophrenia. As with other common disorders, common variants of small effect are being implicated. Some of these variants appear to also contribute risk to other psychiatric phenotypes, in particular bipolar disorder. A point of difference from bipolar disorder, which links schizophrenia to other neurodevelopmental disorders, is the identified contribution of rare CNVs, which collectively may account for some proportion of total susceptibility to schizophrenia within the population. 

It is too early to know whether the contribution of this rare variation is as significant as it is proving to be in autism [35, 43]. However, these data represent a starting point to test new hypotheses using the modern tools of genomics and neuroscience research to make real breakthroughs in our understanding of the molecular mechanisms involved. 

### 3.1 Reshaping How We Define the Disorder

The few pieces of the genetic puzzle that we now have, suggest a radical re-shaping of the clinical boundaries that define the disorder may be required. At a genetic level schizophrenia overlaps with both adult and childhood disorders of a neurodevelopmental aetiology. Knowing this allows novel studies to assess the biological underpinnings of these clinical entities. Further studies of common variation, for example, using polygene analyses and cross-disorder meta-analyses (currently underway within the Psychiatric GWAS Consortium) will clarify the extent of this overlap. The range of phenotypes associated with known rare variants suggests that its reasonable to extend GWAS studies of common variation to include related medical phenotypes (e.g. seizure disorder). This approach may not go far enough. As previously discussed, a striking number of different biological causes can lead to schizophrenia symptoms; this may involve many brain circuits. The discovery of rare, highly penetrant mutations makes feasible a reverse approach, where biological dissection of these mutations, rather than heterogeneous clinical phenotypes can shape understanding of causation for at least a subset of patients currently defined as having schizophrenia. 

### 3.2 Larger, Bigger, Better?

The Psychiatric GWAS Consortium is now trying to extend its sample to increase power to detect more modest genetic effects. The pace of gene discovery so far is typical of complex disease [44] and data from other disorders suggests that, if successful in doubling the current sample (to >40,000 cases), this is likely to generate many new loci [45]. An obvious question is what can be learned from genetic variants that individually increase risk of the disorder from 1 to 1.1%? The endpoint of GWAS studies is the discovery of biological pathways underlying complex disorders, rather than individual risk loci. For other disorders and traits, including inflammatory bowel disease and body mass, confirmed risk genes fall into specific molecular pathways which extend our understanding of the genetics and biology of these traits [46-47]. Arguably this is already happening in schizophrenia where both the micro-RNA *MIR137 *and zinc finger protein *ZNF804A* genes appear to be involved in regulating the function of other genes. *MIR137* is a particularly promising example, as it is implicated in the regulation of adult neurogenesis [48] and four of the eleven loci identified in the PGC meta-analyses of schizophrenia and bipolar disorder are predicted *MIR137* target [49].

Having better estimates of small effect sizes will also be useful for molecular pathway based analyses of the GWAS data [50]. In principle, jointly examining whether a group of related genes in a functional pathway are associated with a disease may be more powerful than testing individual markers. Before GWAS studies, this approach was hampered by our limited understanding of biology: candidate genes for analysis stemmed from existing hypotheses (e.g. of glutamatergic dysfunction), leading analyses to the circular conclusion that ‘enrichment’ for association within these genes confirmed the hypothesis. A number of different pathway based methods have been applied to schizophrenia GWAS data [51-53]. Although these studies overlapped in the samples examined, with the exception of pathways involved in cell adhesion [51, 53], there is little agreement as to which pathways are being implicated. This may reflect differences in methodology, including different pathways being examined, but is also likely to reflect ‘noise’ in the data due to the many false positive findings in smaller datasets, where measures of individual risk at a SNP-level are imprecise. If this is the case, testing with larger samples may provide more consistent signals and be valuable in improving pathway annotation. Having better estimates of common, small genetic risk effects may also address a second important question. If there are a large number of common risk variants within the population, does the total number of variants carried by an individual predict risk or outcome for the disorder? A question that could be addressed by modelling total risk SNP burden. 

### 3.3 Smaller, Better, Best?

From a recent review of the emerging CNV literature <5% of schizophrenia patients carry at least one of the risk CNVs identified to date [35]. Assessing their pathogenic significance is still a challenge (this issue is discussed more fully in Lee & Scherer, 2010 [54]). For instance, recurrent CNVs involving the putative schizophrenia risk genes *NRXN1* and *erbB4* are reported in control populations. More information on the prevalence in the general population and the phenotypic consequences of carrying these mutations for diagnosis and prognosis is urgently required. With the exception of 22q11.2DS we know little about their clinical features [28]. Some of these mutations may have a core of shared phenotypic features, as is the case with certain ASDs (e.g. Prader-Willi/Angelman syndrome) but others may have a wide range of phenotypic effects. 

Whole genome sequencing data will become available for hundreds, if not thousands of schizophrenia patients in the next couple of years. Sampling all classes of rare genetic mutations may substantially increase our estimation of how important rare mutations are in the aetiology of schizophrenia. Based on deep re-sequencing data, a recent study suggests that there is an increased rate of potentially deleterious *de novo* mutations in schizophrenia and autism patients [55]. Attaching pathogenic significance to rare, or even private, point mutations will be even more challenging than for CNVs. The likely first step will be to assess the full spectrum of potentially causative variants at known common (e.g. *ZNF804A* and *TCF4*) and rare risk genes (e.g. *VIPR2* and *NRXN1*). A second step will be to establish whether implicated genes can be logically grouped based on their biology, and whether other genes in these pathways also harbor risk mutations (as was the case for *DISC1 *[56]). This will bring into focus much smaller, more detailed studies to assess the impact of particular mutations within carriers and their families, to establish if they are inherited or are occurring *de novo* and assessment of whether mutations can be grouped, based on biology, for phenotypic or pharmacogenetics studies. 

Other study designs may also be informative. One possibility being to sample families affected by a ‘syndromal’ form of schizophrenia who have additional phenotypes including learning disability, autism, seizure disorder or other developmental difficulties. This approach has been remarkably successful for severe neurodevelopmental disorders, where a genetic cause is identifiable in more than 60% of cases [57]. We can learn several lessons from these studies. Firstly, mutations that block, or impair formation of full-length proteins have more severe phenotypic consequences. If these types of mutations contribute appreciably to schizophrenia they will be the most easily detected by whole-genome sequencing analysis. Secondly, for any gene there is likely to be a spectrum of mutations within the human population [58]. For genes where null mutations are known to cause severe neurodevelopmental phenotypes affecting gross brain structure (e.g. *DCX*), less deleterious mutations of the same gene are associated with more subtle brain phenotypes [59]. It may be reasonable to test genes known for involvement in severe brain developmental phenotypes for less deleterious mutations, which could be relevant to schizophrenia or psychosis. As an example, Pitt-Hopkins Syndrome (PTHS), which is a developmental disorder with severe learning disability, can be caused by haploinsufficiency of *TCF4* or deletions/missense mutations of *NRXN1*. Both of these genes are implicated in schizophrenia [60].

### 3.4 One or Many Diseases? 

**Fig. (3):**
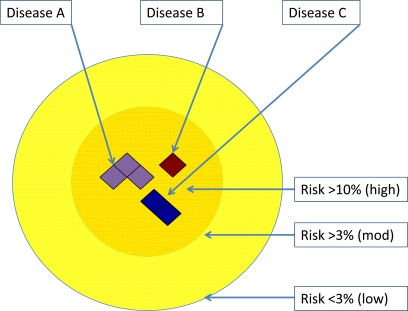
The identification of rare mutations may allow more detailed stratification of psychosis in ‘at risk’ families by the penetrance of these mutations. At highest risk may be individuals with genomic disorders that significantly elevate risk (Disease B e.g. 22q11.2 deletion syndrome), but this may include carriers of multiple mutations impacting particular molecular processes (in the examples of Disease A or C).

At this point we do not know whether the term ‘schizophrenia’ captures one or many different diseases. We have identified common risk variants, which may implicate many different brain circuits and predispose to psychosis through one or many different mechanisms involving information processing or salience i.e. one’s ability to attend to the most relevant or important aspect of available sensory information. Is this a generic psychosis risk for some or all of the disorders that predispose to psychosis (in Fig. **1**)? The identification of rare variants with much larger effects on individual risk raises further questions. Do the deletions at *VIPR2 *and *NRXN1* represent entirely different diseases? If so, do they implicate different signaling pathways or do they converge on a common molecular mechanism, for example, involving GSK3ß signaling? Future models of psychotic disorder may be defined by genetic risk, where groups with higher rates of risk will be identified and possibly defined as having distinct diseases at a molecular level (e.g. Fig. **3**).

## CONCLUSION

The modern research toolbox allows mutations to be studied using approaches that can address these questions. For example, as demonstrated by Brennand and colleagues [61], human cellular models of schizophrenia are increasingly feasible. They have reported on a cellular model of schizophrenia generated using neuron–type cells generated using human induced pluripotent stem cell (hiPSC) technology from schizophrenia patients. Combined with gene expression profiling this identified altered expression of many components of the cyclic AMP and WNT signaling pathways in a small number of patients. As the authors acknowledge, this analysis assumes that schizophrenia is one disease. Applying this approach using patients grouped by mutation is a potentially powerful way of addressing the question of whether ‘schizophrenia’ involves one or many disease processes. Modern imaging methods make it possible to examine neural circuitry, using diffusion tensor imaging, in these same subjects. In parallel, these same mutations can be modeled directly in animal systems to generate cellular phenotypes and investigate neural circuits *in vivo *using viral tracing [62] and optogenetics [63] methods*. *The application of these approaches, with better models, promises new insights into molecular aetiology and potentially novel therapeutics. From some future perspective the diagnosis and treatment of neurodevelopmental disorders based on clinical symptomatology may appear shocking indeed. 

## Figures and Tables

**Table 1.** Genome-Wide Significant Schizophrenia Loci

Chr	Gene Name	SNP	Reference
1p21.3	microRNA 137 (*MIR137*)	rs1625579	[23, 26]
2q32	zinc finger protein 804A (*ZNF804A*)	rs1344706	[22]
2q32.3	prostate-specific transcript 1 (*PCGEM1*)	rs17662626	[26]
6p21-6p22	major histocompatibility complex (*MHC*)	rs6913660 rs6932590 rs13211507 rs3131296	[24]
8p21.3	matrix metallopeptidase 16 (*MMP16*)	rs7004633	[26]
8p23.2	CUB and Sushi multiple domains 1 (*CSMD1*) gene	rs10503253	[26]
10q24.32	cyclin M2 (*CNNM2*)	rs7914558	[26]
11q24	neurogranin (*NRGN*)	rs12807809	[24]
18q21	transcription factor 4 (*TCF4*)	rs9960767	[24]

## References

[1] Dunlop I (1972). The Shock of the New.

[2] Crow T J (2008). The emperors of the schizophrenia polygene have no clothes. Psychol. Med.

[3] Manolio T A, Collins F S, Cox N J, Goldstein D B, Hindorff L A, Hunter D J, McCarthy M I, Ramos E M, Cardon L R, Chakravarti A, Cho J H, Guttmacher A E, Kong A, Kruglyak L, Mardis E, Rotimi C N, Slatkin M, Valle D, Whittemore A S, Boehnke M, Clark A G, Eichler E E, Gibson G, Haines J L, Mackay T F, McCarroll S A, Visscher P M (2009). Finding the missing heritability of complex diseases. Nature.

[4] Tiihonen J, Lonnqvist J, Wahlbeck K, Klaukka T, Niskanen L, Tanskanen A, Haukka J (2009). 11-year follow-up of mortality in patients with schizophrenia: a population-based cohort study (FIN11 study). Lancet.

[5] (2000). Diagnostic and statistical manual of mental disorders.

[6] Davison K, Bagley CR (1969). Schizophrenia-like psychoses associated with organic disorders of the central nervous system: A review of the literature Current Problems in Neuropsychiatry: Schizophrenia, Epilepsy, the Temporal Lobe. Special Publication No. 4.

[7] Kendler K S, Gallagher T J, Abelson J M, Kessler R C (1996). Lifetime prevalence, demographic risk factors, and diagnostic validity of nonaffective psychosis as assessed in a US community sample. The National Comorbidity Survey. Arch. Gen. Psychiatry.

[8] Morgan V A, Leonard H, Bourke J, Jablensky A (2008). Intellectual disability co-occurring with schizophrenia and other psychiatric illness: population-based study. Br. J. Psychiatry.

[9] Carpenter W T (2010). Conceptualizing schizophrenia through attenuated symptoms in the population. Am. J. Psychiatry.

[10] Cardno A G, Gottesman II (2000). Twin studies of schizophrenia: from bow-and-arrow concordances to star wars Mx and functional genomics. Am. J. Med. Genet.

[11] Cardno A G, Rijsdijk F V, Sham P C, Murray R M, McGuffin P (2002). A twin study of genetic relationships between psychotic symptoms. Am. J. Psychiatry.

[12] Parnas J, Cannon T D, Jacobsen B, Schulsinger H, Schulsinger F, Mednick S A (1993). Lifetime DSM-III-R diagnostic outcomes in the offspring of schizophrenic mothers. Results from the Copenhagen High-Risk Study. Arch. Gen. Psychiatry.

[13] Kraepelin E (1920). Die Erscheinungsformen des Irreseins. [Zeitschrift fr die gesamte Neurologie und Psychiatrie.

[14] Blackwood D H, Fordyce A, Walker M T, St Clair D M, Porteous D J, Muir W J (2001). Schizophrenia and affective disorders--cosegregation with a translocation at chromosome 1q42 that directly disrupts brain-expressed genes: clinical and P300 findings in a family. Am. J. Hum. Genet.

[15] Lichtenstein P, Yip B H, Bjork C, Pawitan Y, Cannon T D, Sullivan P F, Hultman C M (2009). Common genetic determinants of schizophrenia and bipolar disorder in Swedish families: a population-based study. Lancet.

[16] Daniels J L, Forssen U, Hultman C M, Cnattingius S, Savitz D A, Feychting M, Sparen P (2008). Parental psychiatric disorders associated with autism spectrum disorders in the offspring. Pediatrics.

[17] Mortensen P B, Pedersen M G, Pedersen C B (2010). Psychiatric family history and schizophrenia risk in Denmark: which mental disorders are relevant?. Psychol. Med.

[18] Risch N (1990). Genetic linkage and complex diseases, with special reference to psychiatric disorders. Genet. Epidemiol.

[19] McCarthy M I, Abecasis G R, Cardon L R, Goldstein D B, Little J, Ioannidis J P, Hirschhorn J N (2008). Genome-wide association studies for complex traits: consensus, uncertainty and challenges. Nat. Rev. Genet.

[20] http://www.szgene.org/genoverview.asp?geneid=861.

[21] Gill M, Donohoe G, Corvin A (2010). What have the genomics ever done for the psychoses?. Psychol. Med.

[22] O'Donovan M C, Craddock N, Norton N, Williams H, Peirce T, Moskvina V, Nikolov I, Hamshere M, Carroll L, Georgieva L, Dwyer S, Holmans P, Marchini J L, Spencer C C, Howie B, Leung H T, Hartmann A M, Moller H J, Morris D W, Shi Y, Feng G, Hoffmann P, Propping P, Vasilescu C, Maier W, Rietschel M, Zammit S, Schumacher J, Quinn E M, Schulze T G, Williams N M, Giegling I, Iwata N, Ikeda M, Darvasi A, Shifman S, He L, Duan J, Sanders A R, Levinson D F, Gejman P V, Cichon S, Nothen M M, Gill M, Corvin A, Rujescu D, Kirov G, Owen M J, Buccola N G, Mowry B J, Freedman R, Amin F, Black D W, Silverman J M, Byerley W F, Cloninger C R (2008). Identification of loci associated with schizophrenia by genome-wide association and follow-up. Nat. Genet.

[23] Purcell S M, Wray N R, Stone J L, Visscher P M, O'Donovan M C, Sullivan P F, Sklar P (2009). Common polygenic variation contributes to risk of schizophrenia and bipolar disorder. Nature.

[24] Stefansson H, Ophoff R A, Steinberg S, Andreassen O A, Cichon S, Rujescu D, Werge T, Pietilainen O P, Mors O, Mortensen P B, Sigurdsson E, Gustafsson O, Nyegaard M, Tuulio-Henriksson A, Ingason A, Hansen T, Suvisaari J, Lonnqvist J, Paunio T, Borglum A D, Hartmann A, Fink-Jensen A, Nordentoft M, Hougaard D, Norgaard-Pedersen B, Bottcher Y, Olesen J, Breuer R, Moller H J, Giegling I, Rasmussen H B, Timm S, Mattheisen M, Bitter I, Rethelyi J M, Magnusdottir B B, Sigmundsson T, Olason P, Masson G, Gulcher J R, Haraldsson M, Fossdal R, Thorgeirsson T E, Thorsteinsdottir U, Ruggeri M, Tosato S, Franke B, Strengman E, Kiemeney L A, Melle I, Djurovic S, Abramova L, Kaleda V, Sanjuan J, de Frutos R, Bramon E, Vassos E, Fraser G, Ettinger U, Picchioni M, Walker N, Toulopoulou T, Need A C, Ge D, Yoon J L, Shianna K V, Freimer N B, Cantor R M, Murray R, Kong A, Golimbet V, Carracedo A, Arango C, Costas J, Jonsson E G, Terenius L, Agartz I, Petursson H, Nothen M M, Rietschel M, Matthews P M, Muglia P, Peltonen L, St Clair D, Goldstein D B, Stefansson K, Collier D A (2009). Common variants conferring risk of schizophrenia. Nature.

[25] Shi J, Levinson D F, Duan J, Sanders A R, Zheng Y, Pe'er I, Dudbridge F, Holmans P A, Whittemore A S, Mowry B J, Olincy A, Amin F, Cloninger C R, Silverman J M, Buccola N G, Byerley W F, Black D W, Crowe R R, Oksenberg J R, Mirel D B, Kendler K S, Freedman R, Gejman P V (2009). Common variants on chromosome 6p22.1 are associated with schizophrenia. Nature.

[26] Ripke S, Sanders A R, Kendler K S, Levinson D F, Sklar P, Holmans P A, Lin D Y, Duan J, Ophoff R A, Andreassen O A, Scolnick E, Cichon S, St Clair D, Corvin A, Gurling H, Werge T, Rujescu D, Blackwood D H, Pato C N, Malhotra A K, Purcell S, Dudbridge F, Neale B M, Rossin L, Visscher P M, Posthuma D, Ruderfer D M, Fanous A, Stefansson H, Steinberg S, Mowry B J, Golimbet V, De Hert M, Jonsson E G, Bitter I, Pietilainen O P, Collier D A, Tosato S, Agartz I, Albus M, Alexander M, Amdur R L, Amin F, Bass N, Bergen S E, Black D W, Borglum A D, Brown M A, Bruggeman R, Buccola N G, Byerley W F, Cahn W, Cantor R M, Carr V J, Catts S V, Choudhury K, Cloninger C R, Cormican P, Craddock N, Danoy P A, Datta S, de Haan L, Demontis D, Dikeos D, Djurovic S, Donnelly P, Donohoe G, Duong L, Dwyer S, Fink-Jensen A, Freedman R, Freimer N B, Friedl M, Georgieva L, Giegling I, Gill M, Glenthoj B, Godard S, Hamshere M, Hansen M, Hansen T, Hartmann A M, Henskens F A, Hougaard D M, Hultman C M, Ingason A, Jablensky A V, Jakobsen K D, Jay M, Jurgens G, Kahn R S, Keller M C, Kenis G, Kenny E, Kim Y, Kirov G K, Konnerth H, Konte B, Krabbendam L, Krasucki R, Lasseter V K, Laurent C, Lawrence J, Lencz T, Lerer F B, Liang K Y, Lichtenstein P, Lieberman J A, Linszen D H, Lonnqvist J, Loughland C M, Maclean A W, Maher B S, Maier W, Mallet J, Malloy P, Mattheisen M, Mattingsdal M, McGhee K A, McGrath J J, McIntosh A, McLean D E, McQuillin A, Melle I, Michie P T, Milanova V, Morris D W, Mors O, Mortensen P B, Moskvina V, Muglia P, Myin-Germeys I, Nertney D A, Nestadt G, Nielsen J, Nikolov I, Nordentoft M, Norton N, Nothen M M, O'Dushlaine C T, Olincy A, Olsen L, O'Neill F A, Orntoft T F, Owen M J, Pantelis C, Papadimitriou G, Pato M T, Peltonen L, Petursson H, Pickard B, Pimm J, Pulver A E, Puri V, Quested D, Quinn E M, Rasmussen H B, Rethelyi J M, Ribble R, Rietschel M, Riley B P, Ruggeri M, Schall U, Schulze T G, Schwab S G, Scott R J, Shi J, Sigurdsson E, Silverman J M, Spencer C C, Stefansson K, Strange A, Strengman E, Stroup T S, Suvisaari J, Terenius L, Thirumalai S, Thygesen J H, Timm S, Toncheva D, van den Oord E, van Os J, van Winkel R, Veldink J, Walsh D, Wang A G, Wiersma D, Wildenauer D B, Williams H J, Williams N M, Wormley B, Zammit S, Sullivan P F, O'Donovan M C, Daly M J, Gejman P V (2011). Genome-wide association study identifies five new schizophrenia loci. Nat. Genet.

[27] Williams H J, Norton N, Dwyer S, Moskvina V, Nikolov I, Carroll L, Georgieva L, Williams N M, Morris D W, Quinn E M, Giegling I, Ikeda M, Wood J, Lencz T, Hultman C, Lichtenstein P, Thiselton D, Maher B S, Malhotra A K, Riley B, Kendler K S, Gill M, Sullivan P, Sklar P, Purcell S, Nimgaonkar V L, Kirov G, Holmans P, Corvin A, Rujescu D, Craddock N, Owen M J, O'Donovan M C (2011). Fine mapping of ZNF804A and genome-wide significant evidence for its involvement in schizophrenia and bipolar disorder. Mol. Psychiatry.

[28] Karayiorgou M, Simon T J, Gogos J A (2010). 22q11.2 microdeletions: linking DNA structural variation to brain dysfunction and schizophrenia. Nat. Rev. Neurosci.

[29] Lemberg M, Thompson A W (2010). Marfan syndrome and schizophrenia: a case report and literature review. Gen. Hosp. Psychiatry.

[30] Sebat J, Lakshmi B, Troge J, Alexander J, Young J, Lundin P, Maner S, Massa H, Walker M, Chi M, Navin N, Lucito R, Healy J, Hicks J, Ye K, Reiner A, Gilliam T C, Trask B, Patterson N, Zetterberg A, Wigler M (2004). Large-scale copy number polymorphism in the human genome. Science.

[31] Iafrate A J, Feuk L, Rivera M N, Listewnik M L, Donahoe P K, Qi Y, Scherer S W, Lee C (2004). Detection of large-scale variation in the human genome. Nat. Genet.

[32] Walsh T, McClellan J M, McCarthy S E, Addington A M, Pierce S B, Cooper G M, Nord A S, Kusenda M, Malhotra D, Bhandari A, Stray S M, Rippey C F, Roccanova P, Makarov V, Lakshmi B, Findling R L, Sikich L, Stromberg T, Merriman B, Gogtay N, Butler P, Eckstrand K, Noory L, Gochman P, Long R, Chen Z, Davis S, Baker C, Eichler E E, Meltzer P S, Nelson S F, Singleton A B, Lee M K, Rapoport J L, King M C, Sebat J (2008). Rare structural variants disrupt multiple genes in neurodevelopmental pathways in schizophrenia. Science.

[33] Consortium I S (2008). Rare chromosomal deletions and duplications increase risk of schizophrenia. Nature.

[34] Stefansson H, Rujescu D, Cichon S, Pietilainen O P, Ingason A, Steinberg S, Fossdal R, Sigurdsson E, Sigmundsson T, Buizer-Voskamp J E, Hansen T, Jakobsen K D, Muglia P, Francks C, Matthews P M, Gylfason A, Halldorsson B V, Gudbjartsson D, Thorgeirsson T E, Sigurdsson A, Jonasdottir A, Bjornsson A, Mattiasdottir S, Blondal T, Haraldsson M, Magnusdottir B B, Giegling I, Moller H J, Hartmann A, Shianna K V, Ge D, Need A C, Crombie C, Fraser G, Walker N, Lonnqvist J, Suvisaari J, Tuulio-Henriksson A, Paunio T, Toulopoulou T, Bramon E, Di Forti M, Murray R, Ruggeri M, Vassos E, Tosato S, Walshe M, Li T, Vasilescu C, Muhleisen T W, Wang A G, Ullum H, Djurovic S, Melle I, Olesen J, Kiemeney L A, Franke B, Sabatti C, Freimer N B, Gulcher J R, Thorsteinsdottir U, Kong A, Andreassen O A, Ophoff R A, Georgi A, Rietschel M, Werge T, Petursson H, Goldstein D B, Nothen M M, Peltonen L, Collier D A, St Clair D, Stefansson K (2008). Large recurrent microdeletions associated with schizophrenia. Nature.

[35] Sebat J, Levy D L, McCarthy S E (2009). Rare structural variants in schizophrenia: one disorder, multiple mutations, one mutation, multiple disorders. Trends Genet.

[36] Mefford H C, Sharp A J, Baker C, Itsara A, Jiang Z, Buysse K, Huang S, Maloney V K, Crolla J A, Baralle D, Collins A, Mercer C, Norga K, de Ravel T, Devriendt K, Bongers E M, de Leeuw N, Reardon W, Gimelli S, Bena F, Hennekam R C, Male A, Gaunt L, Clayton-Smith J, Simonic I, Park S M, Mehta S G, Nik-Zainal S, Woods C G, Firth H V, Parkin G, Fichera M, Reitano S, Lo Giudice M, Li K E, Casuga I, Broomer A, Conrad B, Schwerzmann M, Raber L, Gallati S, Striano P, Coppola A, Tolmie J L, Tobias E S, Lilley C, Armengol L, Spysschaert Y, Verloo P, De Coene A, Goossens L, Mortier G, Speleman F, van Binsbergen E, Nelen M R, Hochstenbach R, Poot M, Gallagher L, Gill M, McClellan J, King M C, Regan R, Skinner C, Stevenson R E, Antonarakis S E, Chen C, Estivill X, Menten B, Gimelli G, Gribble S, Schwartz S, Sutcliffe J S, Walsh T, Knight S J, Sebat J, Romano C, Schwartz C E, Veltman J A, de Vries B B, Vermeesch J R, Barber J C, Willatt L, Tassabehji M, Eichler E E (2008). Recurrent rearrangements of chromosome 1q21.1 and variable pediatric phenotypes. N. Engl. J. Med.

[37] Brunetti-Pierri N, Berg J S, Scaglia F, Belmont J, Bacino C A, Sahoo T, Lalani S R, Graham B, Lee B, Shinawi M, Shen J, Kang S H, Pursley A, Lotze T, Kennedy G, Lansky-Shafer S, Weaver C, Roeder E R, Grebe T A, Arnold G L, Hutchison T, Reimschisel T, Amato S, Geragthy M T, Innis J W, Obersztyn E, Nowakowska B, Rosengren S S, Bader P I, Grange D K, Naqvi S, Garnica A D, Bernes S M, Fong C T, Summers A, Walters W D, Lupski J R, Stankiewicz P, Cheung S W, Patel A (2008). Recurrent reciprocal 1q21.1 deletions and duplications associated with microcephaly or macrocephaly and developmental and behavioral abnormalities. Nat. Genet.

[38] Williams N M, Zaharieva I, Martin A, Langley K, Mantripragada K, Fossdal R, Stefansson H, Stefansson K, Magnusson P, Gudmundsson O O, Gustafsson O, Holmans P, Owen M J, O'Donovan M, Thapar A (2010). Rare chromosomal deletions and duplications in attention-deficit hyperactivity disorder: a genome-wide analysis. Lancet.

[39] Walters R G, Jacquemont S, Valsesia A, de Smith A J, Martinet D, Andersson J, Falchi M, Chen F, Andrieux J, Lobbens S, Delobel B, Stutzmann F, El-Sayed Moustafa J S, Chevre J C, Lecoeur C, Vatin V, Bouquillon S, Buxton J L, Boute O, Holder-Espinasse M, Cuisset J M, Lemaitre M P, Ambresin A E, Brioschi A, Gaillard M, Giusti V, Fellmann F, Ferrarini A, Hadjikhani N, Campion D, Guilmatre A, Goldenberg A, Calmels N, Mandel J L, Le Caignec C, David A, Isidor B, Cordier M P, Dupuis-Girod S, Labalme A, Sanlaville D, Beri-Dexheimer M, Jonveaux P, Leheup B, Ounap K, Bochukova E G, Henning E, Keogh J, Ellis R J, Macdermot K D, van Haelst M M, Vincent-Delorme C, Plessis G, Touraine R, Philippe A, Malan V, Mathieu-Dramard M, Chiesa J, Blaumeiser B, Kooy R F, Caiazzo R, Pigeyre M, Balkau B, Sladek R, Bergmann S, Mooser V, Waterworth D, Reymond A, Vollenweider P, Waeber G, Kurg A, Palta P, Esko T, Metspalu A, Nelis M, Elliott P, Hartikainen A L, McCarthy M I, Peltonen L, Carlsson L, Jacobson P, Sjostrom L, Huang N, Hurles M E, O'Rahilly S, Farooqi I S, Mannik K, Jarvelin M R, Pattou F, Meyre D, Walley A J, Coin L J, Blakemore A I, Froguel P, Beckmann J S (2010). A new highly penetrant form of obesity due to deletions on chromosome 16p11.2. Nature.

[40] van Bon B W M, Mefford H C, de Vries B B A, Pagon R A, Bird T D, Dolan C R, Stephens K (2010). 15q13.3 Microdeletion. In GeneReviews.

[41] Grozeva D, Kirov G, Ivanov D, Jones I R, Jones L, Green E K, St Clair D M, Young A H, Ferrier N, Farmer A E, McGuffin P, Holmans P A, Owen M J, O'Donovan M C, Craddock N (2010). Rare copy number variants: a point of rarity in genetic risk for bipolar disorder and schizophrenia. Arch. Gen. Psychiatry.

[42] Vacic V, McCarthy S, Malhotra D, Murray F, Chou H H, Peoples A, Makarov V, Yoon S, Bhandari A, Corominas R, Iakoucheva L M, Krastoshevsky O, Krause V, Larach-Walters V, Welsh D K, Craig D, Kelsoe J R, Gershon E S, Leal S M, Dell Aquila M, Morris D W, Gill M, Corvin A, Insel P A, McClellan J, King M C, Karayiorgou M, Levy D L, DeLisi L E, Sebat J (2011). Duplications of the neuropeptide receptor gene VIPR2 confer significant risk for schizophrenia. Nature.

[43] Abrahams B S, Geschwind D H (2010). Connecting genes to brain in the autism spectrum disorders. Arch. Neurol.

[44] Kim Y, Zerwas S, Trace S E, Sullivan P F (2011). Schizophrenia genetics: where next?. Schizophr. Bull.

[45] Lander E S (2011). Initial impact of the sequencing of the human genome. Nature.

[46] Speliotes E K, Willer C J, Berndt S I, Monda K L, Thorleifsson G, Jackson A U, Allen H L, Lindgren C M, Luan J, Magi R, Randall J C, Vedantam S, Winkler T W, Qi L, Workalemahu T, Heid I M, Steinthorsdottir V, Stringham H M, Weedon M N, Wheeler E, Wood A R, Ferreira T, Weyant R J, Segre A V, Estrada K, Liang L, Nemesh J, Park J H, Gustafsson S, Kilpelainen T O, Yang J, Bouatia-Naji N, Esko T, Feitosa M F, Kutalik Z, Mangino M, Raychaudhuri S, Scherag A, Smith A V, Welch R, Zhao J H, Aben K K, Absher D M, Amin N, Dixon A L, Fisher E, Glazer N L, Goddard M E, Heard-Costa N L, Hoesel V, Hottenga J J, Johansson A, Johnson T, Ketkar S, Lamina C, Li S, Moffatt M F, Myers R H, Narisu N, Perry J R, Peters M J, Preuss M, Ripatti S, Rivadeneira F, Sandholt C, Scott L J, Timpson N J, Tyrer J P, van Wingerden S, Watanabe R M, White C C, Wiklund F, Barlassina C, Chasman D I, Cooper M N, Jansson J O, Lawrence R W, Pellikka N, Prokopenko I, Shi J, Thiering E, Alavere H, Alibrandi M T, Almgren P, Arnold A M, Aspelund T, Atwood L D, Balkau B, Balmforth A J, Bennett A J, Ben-Shlomo Y, Bergman R N, Bergmann S, Biebermann H, Blakemore A I, Boes T, Bonnycastle L L, Bornstein S R, Brown M J, Buchanan T A, Busonero F, Campbell H, Cappuccio F P, Cavalcanti-Proenca C, Chen Y D, Chen C M, Chines P S, Clarke R, Coin L, Connell J, Day I N, Heijer M, Duan J, Ebrahim S, Elliott P, Elosua R, Eiriksdottir G, Erdos M R, Eriksson J G, Facheris M F, Felix S B, Fischer-Posovszky P, Folsom A R, Friedrich N, Freimer N B, Fu M, Gaget S, Gejman P V, Geus E J, Gieger C, Gjesing A P, Goel A, Goyette P, Grallert H, Grassler J, Greenawalt D M, Groves C J, Gudnason V, Guiducci C, Hartikainen A L, Hassanali N, Hall A S, Havulinna A S, Hayward C, Heath A C, Hengstenberg C, Hicks A A, Hinney A, Hofman A, Homuth G, Hui J, Igl W, Iribarren C, Isomaa B, Jacobs K B, Jarick I, Jewell E, John U, Jorgensen T, Jousilahti P, Jula A, Kaakinen M, Kajantie E, Kaplan L M, Kathiresan S, Kettunen J, Kinnunen L, Knowles J W, Kolcic I, Konig I R, Koskinen S, Kovacs P, Kuusisto J, Kraft P, Kvaloy K, Laitinen J, Lantieri O, Lanzani C, Launer L J, Lecoeur C, Lehtimaki T, Lettre G, Liu J, Lokki M L, Lorentzon M, Luben R N, Ludwig B, Manunta P, Marek D, Marre M, Martin N G, McArdle W L, McCarthy A, McKnight B, Meitinger T, Melander O, Meyre D, Midthjell K, Montgomery G W, Morken M A, Morris A P, Mulic R, Ngwa J S, Nelis M, Neville M J, Nyholt D R, O'Donnell C J, O'Rahilly S, Ong K K, Oostra B, Pare G, Parker A N, Perola M, Pichler I, Pietilainen K H, Platou C G, Polasek O, Pouta A, Rafelt S, Raitakari O, Rayner N W, Ridderstrale M, Rief W, Ruokonen A, Robertson N R, Rzehak P, Salomaa V, Sanders A R, Sandhu M S, Sanna S, Saramies J, Savolainen M J, Scherag S, Schipf S, Schreiber S, Schunkert H, Silander K, Sinisalo J, Siscovick D S, Smit J H, Soranzo N, Sovio U, Stephens J, Surakka I, Swift A J, Tammesoo M L, Tardif J C, Teder-Laving M, Teslovich T M, Thompson J R, Thomson B, Tonjes A, Tuomi T, van Meurs J B, van Ommen G J, Vatin V, Viikari J, Visvikis-Siest S, Vitart V, Vogel C I, Voight B F, Waite L L, Wallaschofski H, Walters G B, Widen E, Wiegand S, Wild S H, Willemsen G, Witte D R, Witteman J C, Xu J, Zhang Q, Zgaga L, Ziegler A, Zitting P, Beilby J P, Farooqi I S, Hebebrand J, Huikuri H V, James A L, Kahonen M, Levinson D F, Macciardi F, Nieminen M S, Ohlsson C, Palmer L J, Ridker P M, Stumvoll M, Beckmann J S, Boeing H, Boerwinkle E, Boomsma D I, Caulfield M J, Chanock S J, Collins F S, Cupples L A, Smith G D, Erdmann J, Froguel P, Gronberg H, Gyllensten U, Hall P, Hansen T, Harris T B, Hattersley A T, Hayes R B, Heinrich J, Hu F B, Hveem K, Illig T, Jarvelin M R, Kaprio J, Karpe F, Khaw K T, Kiemeney L A, Krude H, Laakso M, Lawlor D A, Metspalu A, Munroe P B, Ouwehand W H, Pedersen O, Penninx B W, Peters A, Pramstaller P P, Quertermous T, Reinehr T, Rissanen A, Rudan I, Samani N J, Schwarz P E, Shuldiner A R, Spector T D, Tuomilehto J, Uda M, Uitterlinden A, Valle T T, Wabitsch M, Waeber G, Wareham N J, Watkins H, Wilson J F, Wright A F, Zillikens M C, Chatterjee N, McCarroll S A, Purcell S, Schadt E E, Visscher P M, Assimes T L, Borecki I B, Deloukas P, Fox C S, Groop L C, Haritunians T, Hunter D J, Kaplan R C, Mohlke K L, O'Connell J R, Peltonen L, Schlessinger D, Strachan D P, van Duijn C M, Wichmann H E, Frayling T M, Thorsteinsdottir U, Abecasis G R, Barroso I, Boehnke M, Stefansson K, North K E, McCarthy M I, Hirschhorn J N, Ingelsson E, Loos R J (2010). Association analyses of 249,796 individuals reveal 18 new loci associated with body mass index. Nat. Genet.

[47] Franke A, McGovern D P, Barrett J C, Wang K, Radford-Smith G L, Ahmad T, Lees C W, Balschun T, Lee J, Roberts R, Anderson C A, Bis J C, Bumpstead S, Ellinghaus D, Festen E M, Georges M, Green T, Haritunians T, Jostins L, Latiano A, Mathew C G, Montgomery G W, Prescott N J, Raychaudhuri S, Rotter J I, Schumm P, Sharma Y, Simms L A, Taylor K D, Whiteman D, Wijmenga C, Baldassano R N, Barclay M, Bayless T M, Brand S, Buning C, Cohen A, Colombel J F, Cottone M, Stronati L, Denson T, De Vos M, D'Inca R, Dubinsky M, Edwards C, Florin T, Franchimont D, Gearry R, Glas J, Van Gossum A, Guthery S L, Halfvarson J, Verspaget H W, Hugot J P, Karban A, Laukens D, Lawrance I, Lemann M, Levine A, Libioulle C, Louis E, Mowat C, Newman W, Panes J, Phillips A, Proctor D D, Regueiro M, Russell R, Rutgeerts P, Sanderson J, Sans M, Seibold F, Steinhart A H, Stokkers P C, Torkvist L, Kullak-Ublick G, Wilson D, Walters T, Targan S R, Brant S R, Rioux J D, D'Amato M, Weersma R K, Kugathasan S, Griffiths A M, Mansfield J C, Vermeire S, Duerr R H, Silverberg M S, Satsangi J, Schreiber S, Cho J H, Annese V, Hakonarson H, Daly M J, Parkes M (2010). Genome-wide meta-analysis increases to 71 the number of confirmed Crohn's disease susceptibility loci. Nat. Genet.

[48] Szulwach K E, Li X, Smrt R D, Li Y, Luo Y, Lin L, Santistevan N J, Li W, Zhao X, Jin P (2010). Cross talk between microRNA and epigenetic regulation in adult neurogenesis. J. Cell Biol.

[49] Ripke S, Gejman PV (2010). on behalf of the Schizophrenia Psychiatric GWAS Consortium (PGC). Genome-wide association in more than 20,000 samples produces robust evidence for replication and defines novel loci for schizophrenia.

[50] Wang K, Li M, Hakonarson H (2010). Analysing biological pathways in genome-wide association studies. Nat. Rev. Genet.

[51] O'Dushlaine C, Kenny E, Heron E, Donohoe G, Gill M, Morris D, Corvin A (2011). Molecular pathways involved in neuronal cell adhesion and membrane scaffolding contribute to schizophrenia and bipolar disorder susceptibility. Mol. Psychiatry.

[52] Jia P, Wang L, Meltzer H Y, Zhao Z (2011). Pathway-based analysis of GWAS datasets: effective but caution required. Int. J. Neuropsychopharmacol.

[53] Weng L, Macciardi F, Subramanian A, Guffanti G, Potkin S G, Yu Z, Xie X (2011). SNP-based pathway enrichment analysis for genome-wide association studies. BMC Bioinformatics.

[54] Lee C, Scherer S W (2010). The clinical context of copy number variation in the human genome. Expert Rev. Mol. Med.

[55] Awadalla P, Gauthier J, Myers R A, Casals F, Hamdan F F, Griffing A R, Cote M, Henrion E, Spiegelman D, Tarabeux J, Piton A, Yang Y, Boyko A, Bustamante C, Xiong L, Rapoport J L, Addington A M, DeLisi J L, Krebs M O, Joober R, Millet B, Fombonne E, Mottron L, Zilversmit M, Keebler J, Daoud H, Marineau C, Roy-Gagnon M H, Dube M P, Eyre-Walker A, Drapeau P, Stone E A, Lafreniere R G, Rouleau G A (2010). Direct measure of the de novo mutation rate in autism and schizophrenia cohorts. Am. J. Hum. Genet.

[56] Millar J K, Pickard B S, Mackie S, James R, Christie S, Buchanan S R, Malloy M P, Chubb J E, Huston E, Baillie G S, Thomson P A, Hill E V, Brandon N J, Rain J C, Camargo L M, Whiting P J, Houslay M D, Blackwood D H, Muir W J, Porteous D J (2005). DISC1 and PDE4B are interacting genetic factors in schizophrenia that regulate cAMP signaling. Science.

[57] Rimoin D, Connor JM, Reed EP, Korf BR, Emery AE (2006). Emery and Rimoin's Principles and Practice of Medical Genetics.

[58] Brenner S (2003). Nobel lecture. Nature's gift to science. Biosci. Rep.

[59] Walsh C A, Engle E C (2010). Allelic diversity in human developmental neurogenetics: insights into biology and disease. Neuron.

[60] Blake D J, Forrest M, Chapman R M, Tinsley C L, O'Donovan M C, Owen M J (2010). TCF4, schizophrenia, and Pitt-Hopkins Syndrome. Schizophr. Bull.

[61] Brennand K J, Simone A, Jou J, Gelboin-Burkhart C, Tran N, Sangar S, Li Y, Mu Y, Chen G, Yu D, McCarthy S, Sebat J, Gage F H (2011). Modelling schizophrenia using human induced pluripotent stem cells. Nature.

[62] Card J P, Kobiler O, McCambridge J, Ebdlahad S, Shan Z, Raizada M K, Sved A F, Enquist L W (2011). Microdissection of neural networks by conditional reporter expression from a Brainbow herpesvirus. Proc. Natl. Acad. Sci. USA.

[63] Deisseroth K (2011). Optogenetics. Nat. Methods.

